# 

**Published:** 1891-10

**Authors:** 


### CONTENTS.

### COMMUNICATIONS.

Scientific Investigation of the Cranium and Jaws .......... 473
President's Address .......... 482
Starved Teeth .......... 488
Bromine as a Disinfectant .......... 490

### PROCEEDINGS.

National Association of Dental Faculties .......... 491
National Association of Dental Examiners .......... 498
Alumni Association of the Philadelphia Dental College .......... 502

### SELECTIONS.

A New Anaesthetic .......... 503
A method of Differentiating Nerve Tissue with Mercuric Chloride .......... 504
Bacterial Poisons .......... 505
The Phonograph in Medicine .......... 506
Liniment for Bruises .......... 507
Syphilis .......... 508
Nitrite of Amyl in Chloroform Poisoning .......... 509
A Good Cough Mixture .......... 510
Dental Caries .......... 510
Thymol Dentifrice .......... 510
Advertising .......... 511
Legalized .......... 513
In Memoriam .......... 514-516
Central Dental Association of New Jersey .......... 514
Resolutions on the deaths of Drs. Wm. H. Atkinson and John Stephan .......... 515

### EDITORIAL.

Specialties in Dental Practice .......... 518
Cleveland, O., Dental Society .......... 521
Acknowledgment .......... 521
Ohio State Dental Meeting .......... 522
Bibliographical .......... 523
Examiners' Meeting .......... 524

